# Supplementary material for: MiRNAs Expression Profiling in Raw264.7 Macrophages after Nfatc1-Knockdown Elucidates Potential Pathways Involved in Osteoclasts Differentiation
Source: Biology (Basel). 2021 Oct 22;10(11):1080. doi: 10.3390/biology10111080 (PMC8614811; doi:10.3390/biology10111080)
Supplement: Supplementary file 1 [file biology-10-01080-s001.zip › biology-1374858-supplementary.pdf]

Table S1. Sequences of the primers used in experiments.

Primer	Forward	Reverse	use
miR-124-3p	5'CACTCGAGGGCATTCACCGCGTGCCTTAG-3'	5'TCGACTAAGGCACGCGGTGAATGCCC TCGAGTGAGCT-3'	cloning in pmirGLO vector
Luc2	5'-GCAAGATCGCCGTGTAATTC-3'		colony PCR
LucR		5'-AGCAGCCAACTCAGCTTCCTTT-3'	colony PCR
miR-124-3p mimic	UAAGGCACGCGGUGAAUGCC		transfection

Figure S1. NFATc1 mRNA and protein expression in RAW264.7 cells transfected with negative control (NC)- and NFATc1-siRNAs, assessed by qPCR and Western blot respectively.

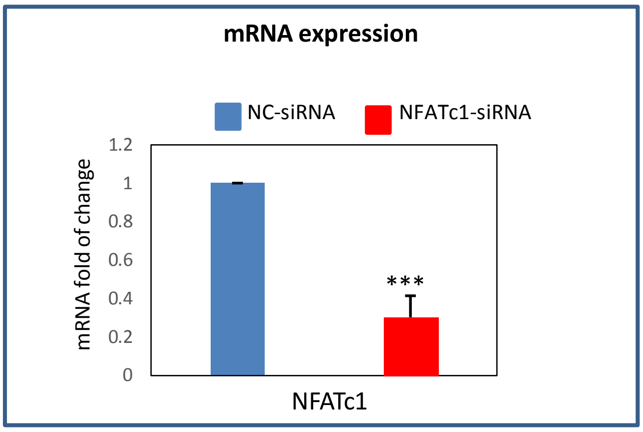

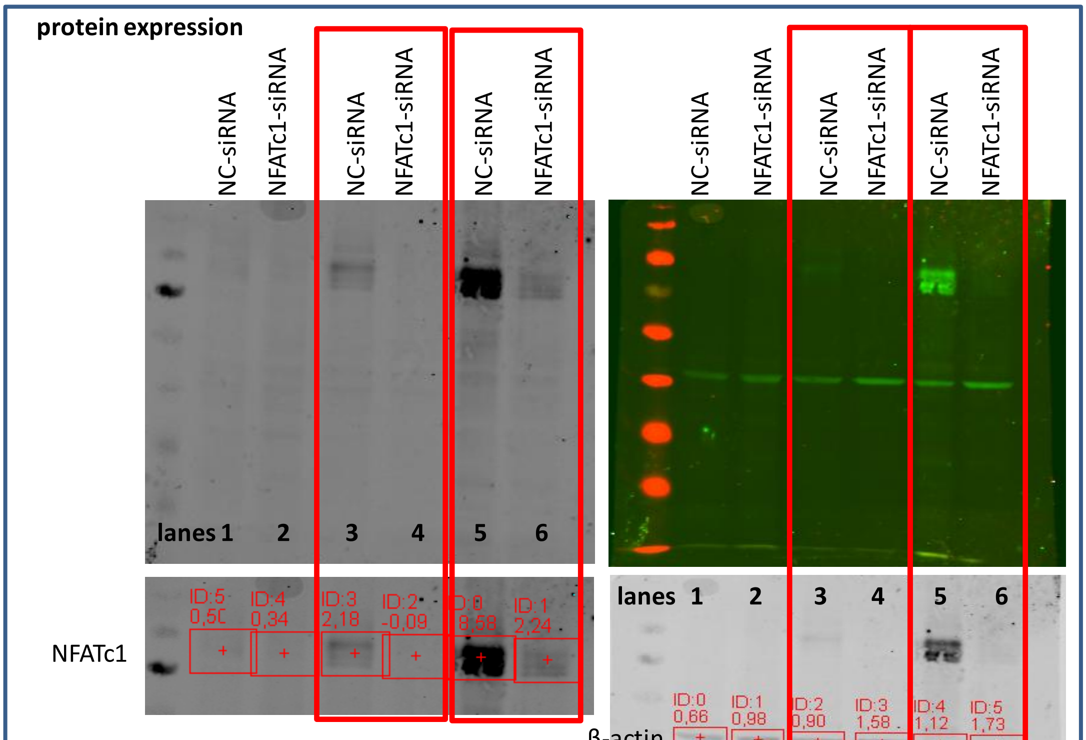

	NFATc1		
lanes		NC-siRNA	NFAT siRNA
3 and 4	NFATc1	2.18	0.09
3 and 4	actin	0.90	1.58
3 and 4	NFATc1/actin	2.42	0.05
	**NFATc1/NC**	**1**	**0.02**
5 and 6	NFATc1	8.58	2.24
5 and 6	actin	1.12	1.73
5 and 6	NFATc1/actin	7.66	1.29
	**NFATc1/NC**	**1**	**0.17**
	**mean**	**1.00**	**0.19**
	**dev st**	**0.00**	**0,12**

Table S2A. KEGG pathway analysis of group 1 miRNAs (10/10)

KEGG pathway	p-value	#genes	#miRNAs
1. Fatty acid biosynthesis (00061)	5.36140822008e-11	3	3
2. Steroid biosynthesis (00100)	1.35867441382e-05	8	3
3. Viral carcinogenesis (05204	1.35867441382e-05	60	6
4. Lysine degradation (00310)	7.65597870255e-05	18	7
**5. Regulation of actin cytoskeleton (04810)**	**0.000242140581318**	**64**	**7**
6. Proteoglycans in cancer (05205)	0.000290127806675	58	9
7. Thyroid hormone signaling pathway (04919)	0.000332301205229	37	6
8. Hepatitis B (005161)	0.000358727205925	40	7
9. Progesterone-mediated oocyte maturation (04914)	0.000595378670869	32	8
10. Bacterial invasion of epithelial cells (05100)	0.000917411337915	26	6
11. N-Glycan biosynthesis (00510)	0.00108179088565	14	6
12. Cell cycle (04510)	0.00108179088565	35	6
13. Axon guidance (04360)	0.00108179088565	38	6
14. Glioma (05214)	0.00108179088565	21	7
15. Prostate cancer (05215)	0.00108179088565	30	7
16. Neurotrophin signaling pathway (04722)	0.00108179088565	40	8
17. Renal cell carcinoma (05211)	0.00108179088565	26	8
18. FoxO signaling pathway (04068)	0.00246198169547	42	8
19. Oocyte meiosis (04114)	0.00434539175804	31	7
20. Long-term potentiation (04720)	0.00434539175804	24	8
21. Endocytosis (04144)	0.00503680676890	54	6
22. Protein processing in endoplasmic reticulum (04141)	0.00503680668229	51	8
23. Adherens junction (04520)	0.00941611189261	23	6
24. Colorectal cancer (05210)	0.00941611189261	20	6
25. mTOR signaling pathway (04150)	0.00941611189261	22	9
26. Estrogen signaling pathway (04915)	0.0101410087705	23	6
27. ErbB signaling pathway (04012)	0.0104964382728	29	7
28. Wnt signaling pathway (04310)	0.0115055779276	38	7
29. Prolactin signaling pathway (04917)	0.0135073367815	22	6
**30. MAPK signaling pathway (04010)**	**0.0135073367815**	**63**	**8**
31. HIF-1 signaling pathway (04066)	0.0154468946498	33	7
32. Pancreatic cancer (05212)	0.0156921463378	21	7
33. Ras signaling pathway (04014)	0.0173657508244	50	8
34. Natural killer cell mediated cytotoxicity (04650)	0.0173657508244	30	8
35. Apoptosis (04210)	0.0201787525489	24	7
36. Chronic myeloid leukemia (05220)	0.021166277299	22	6
37. Pathways in cancer (05230)	0.0224201899433	91	9
**38. Focal adhesion (04510)**	**0.0226092919853**	**53**	**7**
39. HTLV-I infection (05166)	0.0226092919853	68	7
**40. Osteoclast differentiation (04380)**	**0.0254709599574**	**36**	**6**
41. Insulin signaling pathway (04910)	0.0363320441651	38	6
42. Amyotrophic lateral sclerosis (ALS) (05014)	0.0403961787598	17	6
43. Hippo signaling pathway (04390)	0.0403961787598	33	7

44. cGMP-PKG signaling pathway (04022)	0.0403961787598	44	8
45. Gap junction (04540)	0.0450170270892	20	6
46. Phosphatidylinositol signaling system (04070)	0.0491788387378	19	5

Table S2B. KEGG pathway analysis of group 2 miRNAs (28/28)

KEGG pathway	p-value	#genes	#miRNAs
1. Prion diseases (05020)	6.5227867E-10	13	10
2. Proteoglycans in cancer (05205)	6.0461851E-08	96	24
3. Endocytosis (04144)	2.9080974E-07	102	25
4. AMPK signaling pathway (04152)	4.3307460E-06	66	24
**5. Regulation of actin cytoskeleton (04810)**	**8.6120987E-06**	**93**	**24**
6. Adherens junction (04520)	92757187E-06	39	18
7. Renal cell carcinoma (05211)	3.4566814E-05	36	20
8. Phsphatidylinositol signaling system (04070)	0.00014385709	42	17
9. Pathways in cancer (05200)	0.00016003808	153	25
**10. MAPK signaling pathway (04010)**	**0.00017112352**	**105**	**26**
11. Cell cycle (04110)	0.00019967890	58	19
12. GnRH signaling pathway (04912)	0.00025417697	45	22
13. FoxO signaling pathway (04068)	0.00025417697	62	24
14. Thyroid hormone signaling pathway (04919)	0.00041221316	52	21
15. TGF-beta signaling pathway (004350)	0.00041221316	38	22
16. Axon guidance (04360)	0.00041221316	59	22
**17. Focal adhesion (04510)**	**0.00041221316**	**89**	**24**
18. Neutrophin signaling pathway (004722)	0.00041221316	57	25
19. Choline metabolism in cancer (005231)	0.00042283937	49	20
20. Inositol phosphate metabolism (00562)	0.00086189822	28	16
21. Oocyte meiosis (004114)	0.00086189822	50	21
22. Protein processing in endoplasmic reticulum (004141)	0.00086189822	73	23
23. Bacterial invasion of epithelial cells (005100)	0.00150621352	34	19
24. Hippo signaling pathway (004390)	0.00150621352	64	23
25. Prostate cancer (005215)	0.00151965652	42	21
26. Gap junction (004540)	0.00151965652	36	23
27. PI3K-Akt signaling pathway (004151)	0.00116789623	133	26
28. Estrogen signaling pathway (004915)	0.00118281127	42	20
29. Erb signaling pathway (04012)	0.00182893843	39	21
30. mTOR signaling pathway (004150)	0.00256569544	31	18
31. Insulin signaling pathway (004910)	0.00431683687	61	23
32. Glioma (005214)	0.00620105012	28	20
33. Adrenergic signaling in cardiomyocytes (004261)	0.00693163316	58	24
34. Hepatitis B (005161)	0.00839540834	55	20
35. Progesterone-mediated oocyte maturation (004914)	0.00839540834	41	21
36. Oxytocin signaling pathway (04921)	0.00883981551	63	21
37. Arrhythmogenic right ventricular cardiomyopathy (05412)	0.00903399468	34	21
38. Ubiquitin mediated proteolisis (004120)	0.01271797451	62	20
39. Rap1 signaling pathway (004015)	0.01446270968	81	24
40. 2-Oxocarboxylic acid metabolism (001210)	0.01472182954	8	8
41. Pancreatic cancer (005212)	0.01510748641	31	20
42. T cell receptor signaling pathway (004660)	0.01510748641	44	21
43. Colorectal cancer (005210)	0.01542217975	29	21

44. Fc gamma R-mediated phagocytosis (004666)	0.01616154248	32	20
45. Prolactin signaling pathway (004917)	0.01616421548	32	20
46. Salmonella infection (005132)	0.01661874263	34	18
47. Ras signaling pathway (004014)	0.01676152728	81	25
48. Central carbon metabolism in cancer (005230)	0.01898506346	27	18
49. Dilated cardiomyophaty (005414)	0.01949299154	38	23
50. Hypertrophic cardiomyophaty (005410)	0.02188546371	36	22
51. Transcriptional misregulation in cancer (05202)	0.02279832206	65	23
52. Citrate cycle (000020)	0.02353014972	15	11
53. HIF-1 alpha pathway signaling (004066)	0.02397002727	47	18
54. Dorso-ventral axis formation (04320)	0.02461794116	14	18
55. Lysine degradation (00310)	0.03080776531	20	13
56. HTLV-1 infection (05166)	0.03243437970	99	24
57. Amphetamine addiction (05031)	0.03270849842	27	17
**58. Osteoclast differentiation (04380)**	**0.03431825384**	**50**	**23**
59. Acute myeloid leukemia (05221)	0.03565183747	24	18
60. TNF signaling pathway (04668)	0.03565183747	45	22
61. Gastric acid secretion (04971)	0.03593114406	31	16
62. One carbon pool by folate (00670)	0.04061838855	7	6
63. Chagas disease (05142)	0.04061833885	39	20
64. cAMP signaling pathway (004024)	0.04061838855	75	23
65. Steroid biosynthesis (00100)	0.04301718373	7	8
66. Long term depression (04730)	0.04301718373	26	20

Table S2C. KEGG pathway analysis of group 3 miRNAs (6/6)

KEGG pathway	p-value	#genes	#miRNAs
**1. ECM-receptor interaction (04512)**	**2.38994017785e-25**	**4**	**4**
2. Fatty acid biosynthesis (00061)	1.52631231255e-16	4	3
3. Lysine degradation (00310)	1.61410951993e-10	16	3
4. Proteoglycans in cancer (05205)	1.75254998173e-09	51	6
5. Fatty acid metabolism (01212)	2.17918089738e-07	10	3
6. Glioma (005214)	7.68794571091e-06	20	6
7. Oocyte meiosis (04114)	1.19151760973e-05,	30	6
8. Renal cell carcinoma (05211)	2.28545930945e-05	22	6
**9. Focal adhesion (04510)**	**2.43074477409e-05**	**53**	**6**
10. Endocytosis (04144)	3.9260989193e-05	49	6
11. FoxO signaling pathway (04068)	0.000127041152984	35	6
11. Gap junction (04540)	0.000397609828077	20	4
12. ErbB signaling pathway (04012)	0.000451639669319	20	4
13. Transcriptional misregulation in cancer (05202)	0.00060441507812	37	6
14. Progesterone-mediated oocyte maturation (04914)	0.0011148974601	26	6
15. Prostate cancer (05215)	0.00127908177104	24	6
16. mTOR signaling pathway (04150)	0.00135794845682	20	6
17. Pathways in cancer (05200)	0.00137081025218	77	6
18. Neurotrophin signaling pathway (04722)	0.00157519357955	32	5
19. AMPK signaling pathway (04152)	0.0020370687641	29	6
20. PI3K-Akt signaling pathway (04151)	0.00298596894611	69	6
21. Glycosaminoglycan bios.- chond. sulf./ derm. sulf. (00532)	0.00320667175771	4	2
22. Glycosaminoglycan biosynthesis - hep. sulf./ heparin (00534)	0.0037262272458	5	3
23. GnRH signaling pathway (04912)	0.00413394872213	23	4
24. Ras signaling pathway (04014)	0.00460747043151	43	6
25. Steroid biosynthesis (00100)	0.00503370968364	5	1
26. Estrogen signaling pathway (04915)	0.00890741453019	22	5
**27. MAPK signaling pathway (04010)**	**0.0136134307112**	**50**	**6**
28. Rap1 signaling pathway (04015)	0.0150538560358	41	6
29. Long-term potentiation (04720)	0.0155601847529	18	6
30. Cell cycle (04110)	0.0165132896613	26	4
31. Non-small cell lung cancer (05223)	0.0247040002813	16	5
32. Long-term depression (04730)	0.0373838758498	13	3
33. Insulin signaling pathway (04910)	0.0391747801457	30	5
34. Arrhythmogenic right ventricular cardiomyopathy (05412)	0.0409341523648	11	4
35. Colorectal cancer (05210)	0.0481305403395	14	5

Table S2D. KEGG pathway analysis of group 4 miRNAs (4/4).

KEGG pathway	p-value	#genes	#miRNAs
1.Prion diseases (05020)	3.87745724717e-15	8	3
2. Fatty acid metabolism (01212)	2.25175998536e-12	13	3
3. Fatty acid biosynthesis (00061)	6.55652671864e-11	2	2
4. Adherens junction (04520)	2.94982666607e-07	30	3
**5. MAPK signaling pathway (04010)**	**2.61806457963e-05**	**70**	**4**
6. Vitamin B6 metabolism (00750)	4.49524337343e-05	4	3
7. Axon guidance (04360)	4.49524337343e-05	40	4
8. Thyroid hormone signaling pathway (04919)	5.87952248746e-05	39	4
9. Renal cell carcinoma (05211)	0.000119371873751	24	4
10. Lysine degradation (00310)	0.000124527226046	15	4
11. Glioma (05214)	0.000146269820369	22	4
12. FoxO signaling pathway (04068)	0.000183091510842	45	4
13.Protein processing in endoplasmic reticulum (04141)	0.000318724486747	51	4
14. AMPK signaling pathway (04152)	0.000573944102313	40	4
15. HIF-1 signaling pathway (04066)	0.000844222165995	38	4
16. Endocytosis (04144)	0.000876790528935	61	4
17. Hepatitis B (05161)	0.00149977377214	38	4
18. Proteoglycans in cancer (05205)	0.0019216759989	55	4
**19. Regulation of actin cytoskeleton (04810)**	**0.00417318618615**	**57**	**4**
20. Bacterial invasion of epithelial cells (05100)	0.00417318618615	25	4
21. HTLV-I infection (05166)	0.00496583962993	66	4
22. Biosynthesis of unsaturated fatty acids (01040)	0.00506036157971	7	3
23. Prostate cancer (05215)	0.00718943592066	29	4
24. ErbB signaling pathway (04012)	0.00758701108779	29	4
25. Insulin signaling pathway (04910)	0.0108287823726	40	4
26. Ras signaling pathway (04014)	0.0116576072014	51	4
27. mTOR signaling pathway (04150)	0.0151345309413	20	4
28. Chronic myeloid leukemia (05220)	0.01888700259923	22	4
29. T cell receptor signaling pathway (04660)	0.02096215405013	30	4
30. Neurotrophin signaling pathway (04722)	0.0218052848915	34	4
31. Pancreatic cancer (05212)	0.0247015424952	22	4
**32. Focal adhesion (04510)**	**0.0265055523393**	**52**	**4**
33. Synaptic vesicle cycle (04721)	0.0270896343279	17	3
34. Viral carcinogenesis (05203)	0.027200161878	48	4
35. Galactose metabolism (00052)	0.032424353118	7	3
36. Pathways in cancer (05200)	0.0392699197559	86	4

S1A. Group 1 miRNAs involved in selected pathways, showing the number of predicted target genes for each of them, as identified by Tarbase v.8 or MicroT-CDS v.5.0 databases.

	**7/10 miRNAs involved in Regulation of actin cytoskeleton (mmu04810)**	**Genes**		**8/10 miRNAs involved in MAPK signaling pathway (mmu04010)**	**Genes**
**1.**	mmu-miR-30c-5p|Tarbase	28	**1.**	mmu-miR-218-5p|Tarbase	23
**2.**	mmu-miR-218-5p|Tarbase	24	**2.**	mmu-miR-214-3p|Tarbase	6
**3.**	mmu-miR-214-3p|Tarbase	9	**3.**	mmu-miR-467c-5p|Tarbase	7
**4.**	mmu-miR-467c-5p|Tarbase	8	**4.**	mmu-miR-30c-5p|Tarbase	33
**5.**	mmu-miR-29b-3p|Tarbase	15	**5.**	mmu-miR-29b-3p|Tarbase	10
**6.**	mmu-miR-541-5p|microT-CDS	2	**6.**	mmu-miR-488-3p|microT-CDS	7
**7.**	mmu-miR-488-3p|microT-CDS	3	**7.**	mmu-miR-411-5p|microT-CDS	3
			**8.**	mmu-miR-541-5p|microT-CDS	1
	**7/10 miRNAs involved in Focal adhesion (mmu04510)**	**Genes**		**6/10 miRNAs involved in Osteoclast differentiation (mmu04380)**	**Genes**
**1.**	mmu-miR-29b-3p|Tarbase	14	**1.**	mmu-miR-30c-5p|Tarbase	20
**2.**	mmu-miR-30c-5p|Tarbase	21	**2.**	mmu-miR-218-5p|Tarbase	11
**3.**	mmu-miR-218-5p|Tarbase	18	**3.**	mmu-miR-29b-3p|Tarbase	6
**4.**	mmu-miR-488-3p|microT-CDS	4	**4.**	mmu-miR-488-3p|microT-CDS	4
**5.**	mmu-miR-467c-5p|Tarbase	6	**5.**	mmu-miR-214-3p|Tarbase	7
**6.**	mmu-miR-214-3p|Tarbase	8	**6.**	mmu-miR-467c-5p|Tarbase	7
**7.**	mmu-miR-541-5p|microT-CDS	1			

S1B. Group 2 miRNAs involved in selected pathways, showing the number of predicted target genes for each of them, as identified by Tarbase v.8 or MicroT-CDS v.5.0 databases.

<table>
<tr><th></th><th>26/28 miRNA involved in MAPK signaling (mmu04010)</th><th>Genes</th><th></th><th>24/28 miRNA involved in Focal adhesion (mmu04510)</th><th>Genes</th></tr>
<tr><td>1.</td><td>mmu-miR-19b-3p|Tarbase</td><td>32</td><td>1.</td><td>mmu-miR-182-5p|microT-CDS</td><td>10</td></tr>
<tr><td>2.</td><td>mmu-miR-96-5p|microT-CDS</td><td>19</td><td>2.</td><td>mmu-miR-295-3p|microT-CDS</td><td>7</td></tr>
<tr><td>3.</td><td>mmu-miR-15b-5p|microT-CDS</td><td>7</td><td>3.</td><td>mmu-miR-19b-3p|Tarbase</td><td>32</td></tr>
<tr><td>4.</td><td>mmu-miR-144-3p|microT-CDS</td><td>12</td><td>4.</td><td>mmu-miR-10a-5p|microT-CDS</td><td>2</td></tr>
<tr><td>5.</td><td>mmu-miR-182-5p|microT-CDS</td><td>13</td><td>5.</td><td>mmu-miR-96-5p|microT-CDS</td><td>12</td></tr>
<tr><td>6.</td><td>mmu-miR-32-5p|microT-CDS</td><td>10</td><td>6.</td><td>mmu-miR-15b-5p|microT-CDS</td><td>10</td></tr>
<tr><td>7.</td><td>mmu-miR-9-5p|microT-CDS</td><td>24</td><td>7.</td><td>mmu-miR-126a-3p|TargetScan</td><td>1</td></tr>
<tr><td>8.</td><td>mmu-miR-21a-5p|microT-CDS</td><td>8</td><td>8.</td><td>mmu-miR-32-5p|microT-CDS</td><td>16</td></tr>
<tr><td>9.</td><td>mmu-miR-29a-3p|Tarbase</td><td>10</td><td>9.</td><td>mmu-miR-29a-3p|Tarbase</td><td>14</td></tr>
<tr><td>10.</td><td>mmu-miR-23b-3p|Tarbase</td><td>21</td><td>10.</td><td>mmu-miR-23b-3p|Tarbase</td><td>13</td></tr>
<tr><td>11.</td><td>mmu-miR-10a-5p|microT-CDS</td><td>3</td><td>11.</td><td>mmu-miR-880-3p|microT-CDS</td><td>2</td></tr>
<tr><td>12.</td><td>mmu-miR-295-3p|microT-CDS</td><td>14</td><td>12.</td><td>mmu-miR-9-5p|microT-CDS</td><td>15</td></tr>
<tr><td>13.</td><td>mmu-miR-22-5p|microT-CDS</td><td>7</td><td>13.</td><td>mmu-miR-425-5p|microT-CDS</td><td>3</td></tr>
<tr><td>14.</td><td>mmu-miR-196b-5p|microT-CDS</td><td>5</td><td>14.</td><td>mmu-miR-22-5p|microT-CDS</td><td>5</td></tr>
<tr><td>15.</td><td>mmu-miR-196a-5p|microT-CDS</td><td>5</td><td>15.</td><td>mmu-miR-21a-5p|microT-CDS</td><td>4</td></tr>
<tr><td>16.</td><td>mmu-miR-183-5p|TargetScan</td><td>2</td><td>16.</td><td>mmu-miR-142a-3p|microT-CDS</td><td>6</td></tr>
<tr><td>17.</td><td>mmu-miR-142a-3p|microT-CDS</td><td>5</td><td>17.</td><td>mmu-miR-146a-5p|Tarbase</td><td>6</td></tr>
<tr><td>18.</td><td>mmu-miR-146a-5p|Tarbase</td><td>6</td><td>18.</td><td>mmu-miR-144-3p|microT-CDS</td><td>8</td></tr>
<tr><td>19.</td><td>mmu-miR-425-5p|microT-CDS</td><td>1</td><td>19.</td><td>mmu-miR-196b-5p|microT-CDS</td><td>6</td></tr>
<tr><td>20.</td><td>mmu-miR-31-5p|TargetScan</td><td>2</td><td>20.</td><td>mmu-miR-196a-5p|microT-CDS</td><td>7</td></tr>
<tr><td>21.</td><td>mmu-miR-140-5p|Tarbase</td><td>3</td><td>21.</td><td>mmu-miR-28c|microT-CDS</td><td>2</td></tr>
<tr><td>22.</td><td>mmu-miR-335-5p|TargetScan</td><td>2</td><td>22.</td><td>mmu-miR-140-5p|Tarbase</td><td>1</td></tr>
<tr><td>23.</td><td>mmu-miR-880-3p|microT-CDS</td><td>2</td><td>23.</td><td>mmu-miR-31-5p|TargetScan</td><td>1</td></tr>
<tr><td>24.</td><td>mmu-miR-10b-5p|TargetScan</td><td>1</td><td>24.</td><td>mmu-miR-467e-5p|microT-CDS</td><td>1</td></tr>
<tr><td>25.</td><td>mmu-miR-28c|microT-CDS</td><td>3</td><td></td><td></td><td></td></tr>
<tr><td>26.</td><td>mmu-miR-467e-5p|microT-CDS</td><td>1</td><td></td><td></td><td></td></tr>
<tr><td></td><td></td><td></td><td></td><td></td><td></td></tr>
<tr><td></td><td></td><td></td><td></td><td></td><td></td></tr>
<tr><th></th><th>23/28 miRNA involved in Osteoclasts differentiation (mmu04380)</th><th>Genes</th><th></th><th>24/28 miRNA involved in Regulation of actin cytoskeleton (mmu04810)</th><th>Genes</th></tr>
<tr><td>1.</td><td>mmu-miR-19b-3p|Tarbase</td><td>22</td><td>1.</td><td>mmu-miR-23b-3p|Tarbase</td><td>22</td></tr>
<tr><td>2.</td><td>mmu-miR-10a-5p|microT-CDS</td><td>4</td><td>2.</td><td>mmu-miR-19b-3p|Tarbase</td><td>30</td></tr>
<tr><td>3.</td><td>mmu-miR-144-3p|microT-CDS</td><td>5</td><td>3.</td><td>mmu-miR-425-5p|microT-CDS</td><td>1</td></tr>
<tr><td>4.</td><td>mmu-miR-15b-5p|microT-CDS</td><td>7</td><td>4.</td><td>mmu-miR-21a-5p|microT-CDS</td><td>6</td></tr>
<tr><td>5.</td><td>mmu-miR-146a-5p|Tarbase</td><td>1</td><td>5.</td><td>mmu-miR-29a-3p|Tarbase</td><td>15</td></tr>
<tr><td>6.</td><td>mmu-miR-28c|microT-CDS</td><td>2</td><td>6.</td><td>mmu-miR-15b-5p|microT-CDS</td><td>7</td></tr>
<tr><td>7.</td><td>mmu-miR-23b-3p|Tarbase</td><td>12</td><td>7.</td><td>mmu-miR-295-3p|microT-CDS</td><td>8</td></tr>
<tr><td>8.</td><td>mmu-miR-96-5p|microT-CDS</td><td>8</td><td>8.</td><td>mmu-miR-183-5p|TargetScan</td><td>1</td></tr>
<tr><td>9.</td><td>mmu-miR-182-5p|microT-CDS</td><td>7</td><td>9.</td><td>mmu-miR-142a-3p|microT-CDS</td><td>10</td></tr>
<tr><td>10.</td><td>mmu-miR-295-3p|microT-CDS</td><td>4</td><td>10.</td><td>mmu-miR-140-5p|Tarbase</td><td>2</td></tr>
<tr><td>11.</td><td>mmu-miR-9-5p|microT-CDS</td><td>6</td><td>11.</td><td>mmu-miR-144-3p|microT-CDS</td><td>3</td></tr>
<tr><td>12.</td><td>mmu-miR-142a-3p|microT-CDS</td><td>3</td><td>12.</td><td>mmu-miR-182-5p|microT-CDS</td><td>15</td></tr>
<tr><td>13.</td><td>mmu-miR-140-5p|Tarbase</td><td>4</td><td>13.</td><td>mmu-miR-10a-5p|microT-CDS</td><td>2</td></tr>
<tr><td>14.</td><td>mmu-miR-32-5p|microT-CDS</td><td>4</td><td>14.</td><td>mmu-miR-10b-5p|TargetScan</td><td>1</td></tr>
<tr><td>15.</td><td>mmu-miR-425-5p|microT-CDS</td><td>2</td><td>15.</td><td>mmu-miR-467e-5p|microT-CDS</td><td>1</td></tr>
<tr><td>16.</td><td>mmu-miR-22-5p|microT-CDS</td><td>3</td><td>16.</td><td>mmu-miR-126a-3p|TargetScan</td><td>1</td></tr>
<tr><td>17.</td><td>mmu-miR-10b-5p|TargetScan</td><td>1</td><td>17.</td><td>mmu-miR-22-5p|microT-CDS</td><td>2</td></tr>
<tr><td>18.</td><td>mmu-miR-29a-3p|Tarbase</td><td>6</td><td>18.</td><td>mmu-miR-146a-5p|Tarbase</td><td>9</td></tr>
<tr><td>19.</td><td>mmu-miR-467e-5p|microT-CDS</td><td>1</td><td>19.</td><td>mmu-miR-196b-5p|microT-CDS</td><td>7</td></tr>
<tr><td>20.</td><td>mmu-miR-335-5p|TargetScan</td><td>1</td><td>20.</td><td>mmu-miR-196a-5p|microT-CDS</td><td>6</td></tr>
<tr><td>21.</td><td>mmu-miR-21a-5p|microT-CDS</td><td>1</td><td>21.</td><td>mmu-miR-96-5p|microT-CDS</td><td>22</td></tr>
<tr><td>22.</td><td>mmu-miR-196b-5p|microT-CDS</td><td>1</td><td>22.</td><td>mmu-miR-32-5p|microT-CDS</td><td>10</td></tr>
<tr><td>23.</td><td>mmu-miR-196a-5p|microT-CDS</td><td>1</td><td>23.</td><td>mmu-miR-9-5p|microT-CDS</td><td>17</td></tr>
<tr><td></td><td></td><td></td><td>24.</td><td>mmu-miR-880-3p|microT-CDS</td><td>2</td></tr>
</table>

S1C. Group 3 miRNAs involved in selected pathways, showing the number of predicted target genes for each of them, as identified by Tarbase v.8 or MicroT-CDS v.5.0 databases.

	**4/6 miRNA involved in ECM-receptor interaction (mmu04512)**	**Genes**		**6/6 miRNA involved in Focal adhesion (mmu04510)**	**Genes**
**1.**	mmu-miR-29b-3p | Tarbase	19	**1.**	mmu-miR-29b-3p | Tarbase	44
**2.**	mmu-miR-302d-3p | microT-CDS	3	**2.**	mmu-miR-302d-3p | microT-CDS	5
**3.**	mmu-miR-295-3p | microT-CDS	2	**3.**	mmu-miR-295-3p | microT-CDS	7
**4.**	mmu-miR-880-3p | microT-CDS	1	**4.**	mmu-miR-488-3p | microT-CDS	4
			**5.**	mmu-miR-141-3p | Tarbase	2
			**6.**	mmu-miR-880-3p | microT-CDS	2
	**6/6 miRNA involved in MAPK signaling pathway (mmu04010)**	**Genes**			
**1.**	mmu-miR-488-3p | microT-CDS	7			
**2.**	mmu-miR-29b-3p | Tarbase	30			
**3.**	mmu-miR-302d-3p | microT-CDS	11			
**4.**	mmu-miR-295-3p | microT-CDS	5			
**5.**	mmu-miR-141-3p | microT-CDS	6			
**6.**	mmu-miR-880-3p | microT-CDS	2			

S1D. Group 4 miRNAs involved in selected pathways, showing the number of predicted target genes for each of them, as identified by Tarbase v.8 or MicroT-CDS v.5.0 databases.

<table>
<tr><th></th><th>4/4 miRNA involved in MAPK pathway (mmu04010)</th><th>Genes</th><th></th><th>4/4 miRNA involved in Adherens junction (mmu04520)</th><th>Genes</th></tr>
<tr><td>1.</td><td>mmu-let-7i-5p|Tarbase</td><td>45</td><td>1.</td><td>mmu-miR-138-5p|Tarbase</td><td>6</td></tr>
<tr><td>2.</td><td>mmu-miR-23a-3p|Tarbase</td><td>21</td><td>2.</td><td>mmu-let-7i-5p|Tarbase</td><td>19</td></tr>
<tr><td>3.</td><td>mmu-miR-291a-3p|microT-CDS</td><td>14</td><td>3.</td><td>mmu-miR-23a-3p|Tarbase</td><td>7</td></tr>
<tr><td>4.</td><td>mmu-miR-138-5p|Tarbase</td><td>8</td><td>4.</td><td>mmu-miR-291a-3p|microT-CDS</td><td>1</td></tr>
<tr><td></td><td></td><td></td><td></td><td></td><td></td></tr>
<tr><td></td><td></td><td></td><td></td><td></td><td></td></tr>
<tr><th></th><th>4/4 miRNA involved in Regulation of actin cytoskeleton (mmu04810)</th><th>Genes</th><th></th><th>4/4 miRNA involved in Focal Adhesion (mmu04510)</th><th>Genes</th></tr>
<tr><td>1.</td><td>mmu-miR-291a-3p|microT-CDS</td><td>9</td><td>1.</td><td>mmu-let-7i-5p|Tarbase</td><td>34</td></tr>
<tr><td>2.</td><td>mmu-miR-23a-3p|Tarbase</td><td>22</td><td>2.</td><td>mmu-miR-138-5p|Tarbase</td><td>14</td></tr>
<tr><td>3.</td><td>mmu-miR-138-5p|Tarbase</td><td>10</td><td>3.</td><td>mmu-miR-23a-3p|Tarbase</td><td>13</td></tr>
<tr><td>4.</td><td>mmu-let-7i-5p|Tarbase</td><td>30</td><td>4.</td><td>mmu-miR-291a-3p|microT-CDS</td><td>6</td></tr>
</table>

S2A. Lists of genes involved in MAPK pathway (mmu04010) as predicted targets of the Group 1 miRNAs

	Gene Name	Gene Ensembl id		Gene Name	Gene Ensembl id
1.	Fgfr3	ENSMUSG00000054252	33.	Mapk9	ENSMUSG00000020366
2.	Mapk14	ENSMUSG00000053436	34.	Tgfbr2	ENSMUSG00000032440
3.	Rasgrp1	ENSMUSG00000027347	35.	Nras	ENSMUSG00000027852
4.	Rasa2	ENSMUSG00000032413	36.	Rac1	ENSMUSG00000001847
5.	Mef2c	ENSMUSG00000005583	37.	Sos1	ENSMUSG00000024241
6.	Pdgfrb	ENSMUSG00000024620	38.	Rap1b	ENSMUSG00000052681
7.	Tnfrsf1a	ENSMUSG00000030341	39.	Prkaca	ENSMUSG00000005469
8.	Rps6ka6	ENSMUSG00000025665	40.	Rras2	ENSMUSG00000055723
9.	Rap1a	ENSMUSG00000068798	41.	Rasa1	ENSMUSG00000021549
10.	Rps6ka5	ENSMUSG00000021180	42.	Cdc25b	ENSMUSG00000027330
11.	Map4k4	ENSMUSG00000026074	43.	Crk	ENSMUSG00000017776
12.	Fgf4	ENSMUSG00000050917	44.	Prkca	ENSMUSG00000050965
13.	Ppm1a	ENSMUSG00000021096	45.	Dusp10	ENSMUSG00000039384
14.	Map2k6	ENSMUSG00000020623	46.	Tnf	ENSMUSG00000024401
15.	Map3k1	ENSMUSG00000021754	47.	Elk4	ENSMUSG00000026436
16.	Dusp2	ENSMUSG00000027368	48.	Map3k5	ENSMUSG00000071369
17.	Il1r1	ENSMUSG00000026072	49.	Stk4	ENSMUSG00000018209
18.	Rps6ka3	ENSMUSG00000031309	50.	Mapk8ip3	ENSMUSG00000024163
19.	Nfatc3	ENSMUSG00000031902	51.	Ppp3r1	ENSMUSG00000033953
20.	Pak1	ENSMUSG00000030774	52.	Ppp3ca	ENSMUSG00000028161
21.	Fasl	ENSMUSG00000000817	53.	Pak2	ENSMUSG00000022781
22.	Casp3	ENSMUSG00000031628	54.	Atf2	ENSMUSG00000027104
23.	Rapgef2	ENSMUSG00000062232	55.	Dusp5	ENSMUSG00000034765
24.	Ntrk2	ENSMUSG00000055254	56.	Fgf2	ENSMUSG00000037225
25.	Nf1	ENSMUSG00000020716	57.	Cdc42	ENSMUSG00000006699
26.	Atf4	ENSMUSG00000042406	58.	Map3k2	ENSMUSG00000024383
27.	Ppp3cb	ENSMUSG00000021816	59.	Tab2	ENSMUSG00000015755
28.	Prkx	ENSMUSG00000035725	60.	Myc	ENSMUSG00000022346
29.	Crkl	ENSMUSG00000006134	61.	Mapk8	ENSMUSG00000021936
30.	Egfr	ENSMUSG00000020122	62.	Ppp5c	ENSMUSG00000003099
31.	Kras	ENSMUSG00000030265	63.	Srf	ENSMUSG00000015605
32.	Braf	ENSMUSG00000002413			

S2B. Lists of genes involved in MAPK pathway (mmu04010) as predicted targets of the Group 2 miRNAs

	Gene Name	Gene Ensembl id		Gene Name	Gene Ensembl id
1.	Tgfb2	ENSMUSG00000039239	54.	Cacna1e	ENSMUSG00000004110
2.	Mapk14	ENSMUSG00000053436	55.	Egfr	ENSMUSG00000020122
3.	Rasgrp1	ENSMUSG00000027347	56.	Ntf5	ENSMUSG00000074121
4.	Sos2	ENSMUSG00000034801	57.	Map2k4	ENSMUSG00000033352
5.	Fgf10	ENSMUSG00000021732	58.	Fgf13	ENSMUSG00000031137
6.	Stk3	ENSMUSG00000022329	59.	Kras	ENSMUSG00000030265
7.	Rasa2	ENSMUSG00000032413	60.	Braf	ENSMUSG00000002413
8.	Stmn1	ENSMUSG00000028832	61.	Mapk9	ENSMUSG00000020366
9.	Mef2c	ENSMUSG00000005583	62.	Tgfbr2	ENSMUSG00000032440
10.	Jun	ENSMUSG00000052684	63.	Mecom	ENSMUSG00000027684
11.	Map3k3	ENSMUSG00000020700	64.	Nras	ENSMUSG00000027852
12.	Pdgfrb	ENSMUSG00000024620	65.	Rac1	ENSMUSG00000001847
13.	Dusp6	ENSMUSG00000019960	66.	Sos1	ENSMUSG00000024241
14.	Map4k3	ENSMUSG00000024242	67.	Cacng2	ENSMUSG00000019146
15.	Max	ENSMUSG00000059436	68.	Rap1b	ENSMUSG00000052681
16.	Rps6ka6	ENSMUSG00000025665	69.	Mras	ENSMUSG00000032470
17.	Rap1a	ENSMUSG00000068798	70.	Map2k7	ENSMUSG00000002948
18.	Rps6ka5	ENSMUSG00000021180	71.	Rras2	ENSMUSG00000055723
19.	Map4k4	ENSMUSG00000026074	72.	Rasa1	ENSMUSG00000021549
20.	Map2k1	ENSMUSG00000004936	73.	Tgfb3	ENSMUSG00000021253
21.	Fgf7	ENSMUSG00000027208	74.	Cdc25b	ENSMUSG00000027330
22.	Ppm1a	ENSMUSG00000021096	75.	Fgf18	ENSMUSG00000057967
23.	Fos	ENSMUSG00000021250	76.	Crk	ENSMUSG00000017776
24.	Map2k6	ENSMUSG00000020623	77.	Prkca	ENSMUSG00000050965
25.	Map3k1	ENSMUSG00000021754	78.	Dusp10	ENSMUSG00000039384
26.	Rps6ka4	ENSMUSG00000024952	79.	Tnf	ENSMUSG00000024401
27.	Rps6ka1	ENSMUSG00000003644	80.	Nfkb1	ENSMUSG00000028163
28.	Fgf12	ENSMUSG00000022523	81.	Mapkapk2	ENSMUSG00000016528
29.	Map2k3	ENSMUSG00000018932	82.	Elk1	ENSMUSG00000009406
30.	Mknk2	ENSMUSG00000020190	83.	Cacna2d2	ENSMUSG00000010066
31.	Cacnb2	ENSMUSG00000057914	84.	Stk4	ENSMUSG00000018209
32.	Dusp2	ENSMUSG00000027368	85.	Fgf9	ENSMUSG00000021974
33.	Cacna2d1	ENSMUSG00000040118	86.	Ppp3r1	ENSMUSG00000033953
34.	Rps6ka3	ENSMUSG00000031309	87.	Traf6	ENSMUSG00000027164
35.	Grb2	ENSMUSG00000059923	88.	Fgfr2	ENSMUSG00000030849
36.	Nfatc3	ENSMUSG00000031902	89.	Prkacb	ENSMUSG00000005034
37.	Map3k4	ENSMUSG00000014426	90.	Arrb1	ENSMUSG00000018909
38.	Pak1	ENSMUSG00000030774	91.	Pla2g4a	ENSMUSG00000056220
39.	Fasl	ENSMUSG00000000817	92.	Flnc	ENSMUSG00000068699
40.	Cacnb1	ENSMUSG00000020882	93.	Mapk11	ENSMUSG00000053137
41.	Cacnb4	ENSMUSG00000017412	94.	Pak2	ENSMUSG00000022781
42.	Casp3	ENSMUSG00000031628	95.	Atf2	ENSMUSG00000027104
43.	Rapgef2	ENSMUSG00000062232	96.	Map3k7	ENSMUSG00000028284
44.	Dusp7	ENSMUSG00000053716	97.	Dusp5	ENSMUSG00000034765
45.	Nlk	ENSMUSG00000017376	98.	Cdc42	ENSMUSG00000006699
46.	Cacna1i	ENSMUSG00000022416	99.	Map3k2	ENSMUSG00000024383
47.	Tgfbr1	ENSMUSG00000007613	100.	Map3k11	ENSMUSG00000004054
48.	Rasgrf1	ENSMUSG00000032356	101.	Tab2	ENSMUSG00000015755
49.	Nf1	ENSMUSG00000020716	102.	Mapk8	ENSMUSG00000021936
50.	Atf4	ENSMUSG00000042406	103.	Zak	ENSMUSG00000004085
51.	Ppm1b	ENSMUSG00000061130	104.	Srf	ENSMUSG00000015605
52.	Dusp16	ENSMUSG00000030203	105.	Dusp8	ENSMUSG00000037887
53.	Cacna1d	ENSMUSG00000015968			

S2C. Lists of genes involved in MAPK pathway (mmu04010) as predicted targets of the Group 3 miRNAs

	Gene Name	Gene Ensembl id		Gene Name	Gene Ensembl id
**1.**	**Fgfr3**	ENSMUSG00000054252	**26.**	**Map2k4**	ENSMUSG00000033352
**2.**	**Mapk14**	ENSMUSG00000053436	**27.**	**Braf**	ENSMUSG00000002413
**3.**	**Rasgrp**	ENSMUSG00000027347	**28.**	**Tgfbr2**	ENSMUSG00000032440
**4.**	**Sos2**	ENSMUSG00000034801	**29.**	**Nras**	ENSMUSG00000027852
**5.**	**Fgf10**	ENSMUSG00000021732	**30.**	**Sos1**	ENSMUSG00000024241
**6.**	**Stk3**	ENSMUSG00000022329	**31.**	**Rap1b**	ENSMUSG00000052681
**7.**	**Mef2c**	ENSMUSG00000005583	**32.**	**Rras2**	ENSMUSG00000055723
**8.**	**Pdgfrb**	ENSMUSG00000024620	**33.**	**Tgfb3**	ENSMUSG00000021253
**9.**	**Rps6ka6**	ENSMUSG00000025665	**34.**	**Tgfb1**	ENSMUSG00000002603
**10.**	**Rap1a**	ENSMUSG00000068798	**35.**	**Cdc25b**	ENSMUSG00000027330
**11.**	**Rps6ka5**	ENSMUSG00000021180	**36.**	**Pdgfb**	ENSMUSG00000000489
**12.**	**Flnb**	ENSMUSG00000025278	**37.**	**Crk**	ENSMUSG00000017776
**13.**	**Tab1**	ENSMUSG00000022414	**38.**	**Prkca**	ENSMUSG00000050965
**14.**	**Map2k6**	ENSMUSG00000020623	**39.**	**Dusp10**	ENSMUSG00000039384
**15.**	**Map3k1**	ENSMUSG00000021754	**40.**	**Elk1**	ENSMUSG00000009406
**16.**	**Rps6ka1**	ENSMUSG00000003644	**41.**	**Stk4**	ENSMUSG00000018209
**17.**	**Mknk2**	ENSMUSG00000020190	**42.**	**Fgf9**	ENSMUSG00000021974
**18.**	**Dusp2**	ENSMUSG00000027368	**43.**	**Ppp3r1**	ENSMUSG00000033953
**19.**	**Il1r1**	ENSMUSG00000026072	**44.**	**Fgfr2**	ENSMUSG00000030849
**20.**	**Rps6ka3**	ENSMUSG00000031309	**45.**	**Cdc42**	ENSMUSG00000006699
**21.**	**Nfatc3**	ENSMUSG00000031902	**46.**	**Map3k2**	ENSMUSG00000024383
**22.**	**Fasl**	ENSMUSG00000000817	**47.**	**Map3k11**	ENSMUSG00000004054
**23.**	**Casp3**	ENSMUSG00000031628	**48.**	**Tab2**	ENSMUSG00000015755
**24.**	**Ppp3cb**	ENSMUSG00000021816	**49.**	**Mapk8**	ENSMUSG00000021936
**25.**	**Egfr**	ENSMUSG00000020122	**50.**	**Srf**	ENSMUSG00000015605

S2D. Lists of genes involved in MAPK pathway (mmu04010) as predicted targets of the Group 4 miRNAs

	Gene Name	Gene Ensembl id		Gene Name	Gene Ensembl id
1	Rasgrp1	ENSMUSG00000027347	36.	Kras	ENSMUSG00000030265
2.	Fgf10	ENSMUSG00000021732	37.	Mapk9	ENSMUSG00000020366
3.	Mapt	ENSMUSG00000018411	38.	Tgfbr2	ENSMUSG00000032440
4.	Rasa2	ENSMUSG00000032413	39.	Nras	ENSMUSG00000027852
5.	Mef2c	ENSMUSG00000005583	40.	Map3k14	ENSMUSG00000020941
6.	Map3k3	ENSMUSG00000020700	41.	Sos1	ENSMUSG00000024241
7.	Pdgfrb	ENSMUSG00000024620	42.	Rras2	ENSMUSG00000055723
8.	Tnfrsf1a	ENSMUSG00000030341	43.	Dusp3	ENSMUSG00000003518
9.	Cacng4	ENSMUSG00000020723	44.	Dusp4	ENSMUSG00000031530
10.	Dusp6	ENSMUSG00000019960	45.	Tgfb1	ENSMUSG00000002603
11.	Map4k3	ENSMUSG00000024242	46.	Chuk	ENSMUSG00000025199
12.	Max	ENSMUSG00000059436	47.	Crk	ENSMUSG00000017776
13.	Rps6ka5	ENSMUSG00000021180	48.	Prkca	ENSMUSG00000050965
14.	Map4k4	ENSMUSG00000026074	49.	Elk4	ENSMUSG00000026436
15.	Cacna2d3	ENSMUSG00000021991	50.	Nfkb1	ENSMUSG00000028163
16.	Map3k1	ENSMUSG00000021754	51.	Stk4	ENSMUSG00000018209
17.	Rps6ka1	ENSMUSG00000003644	52.	Fgf9	ENSMUSG00000021974
18.	Mknk2	ENSMUSG00000020190	53.	Mapk8ip3	ENSMUSG00000024163
19.	Dusp2	ENSMUSG00000027368	54.	Ppp3r1	ENSMUSG00000033953
20.	Rps6ka3	ENSMUSG00000031309	55.	Rac2	ENSMUSG00000033220
21.	Nfatc3	ENSMUSG00000031902	56.	Ppp3ca	ENSMUSG00000028161
22.	Map3k4	ENSMUSG00000014426	57.	Prkacb	ENSMUSG00000005034
23.	Pak1	ENSMUSG00000030774	58.	Mapk11	ENSMUSG00000053137
24.	Casp3	ENSMUSG00000031628	59.	Pak2	ENSMUSG00000022781
25.	Dusp7	ENSMUSG00000053716	60.	Atf2	ENSMUSG00000027104
26.	Nlk	ENSMUSG00000017376	61.	Dusp5	ENSMUSG00000034765
27.	Ntrk2	ENSMUSG00000055254	62.	Fgf2	ENSMUSG00000037225
28.	Tgfbr1	ENSMUSG00000007613	63.	Cdc42	ENSMUSG00000006699
29.	Rasgrf1	ENSMUSG00000032356	64.	Map3k2	ENSMUSG00000024383
30.	Nf1	ENSMUSG00000020716	65.	Map3k11	ENSMUSG00000004054
31.	Atf4	ENSMUSG00000042406	66.	Tab2	ENSMUSG00000015755
32.	Il1a	ENSMUSG00000027399	67.	Dusp1	ENSMUSG00000024190
33.	Crkl	ENSMUSG00000006134	68.	Rras	ENSMUSG00000038387
34.	Egfr	ENSMUSG00000020122	69.	Srf	ENSMUSG00000015605
35.	Map2k4	ENSMUSG00000033352	70.	Dusp8	ENSMUSG00000037887

Table S3. List of the main genes involved in pathways regulated by the differentially expressed miRNAs highlighted in PCR arrays.

GENE NAME	miRNA	miRNA	miRNA	miRNA	miRNA	miRNA	miRNA	miRNA
CREB1	29a-3p	23b-3p	10a-5p	15b-5p	19a-3p	23a-3p	138-5p	124-3p
CREbbp	29b-3p	19a-3p	19b-3p	9-5p	7i-5p	124-3p		
MAP2K3 (MKK3)	29a-3p							
MAP2K6 (p38 k.)	29a-3p	29b-3p						
MAPK14 (p38-α)	31-5p	140-5p	29a-3p	29b-3p	141-3p	124-3p		
MAPK11 (p38-β)	335-5p	29a-3p	29b-3p	7i-5p				
MAPK12 (p38-γ)	29a-3p	29b-3p						
MAPK13 (p38-δ)	29a-3p	29b-3p						
MAPK8 (JNK1)	335-5p	196a-5p	19a-3p	19b-3p	31-5p	29a-3p	29b-3p	
MAPK9 (JNK2)	31-5p	7i-5p						
MAPK10 (JNK3)	9-5p							
MAP3K1 (MEKK1)	31-5p	23a-3p	291a-3p					
MAP3K2 (MEKK2)	295-3p	295-3p	141-3p	23a-3p	291a-3p	124-3p		
MAP2K1 (MEK1)	22-3p							
MAPK3 (ERK1)	15b-5p							
MAPK1 (ERK2)	141-3p							
DUSP1	411-5p	144-3p	31-5p	7i-5p				
DUSP2	295-3p	880-3p	880-3p	295-3p	291a-3p			
DUSP3	7i-5p	124-3p						
DUSP5	7i-5p	23a-3p						
DUSP6	7i-5p							
DUSP7	31-5p	7i-5p						
DUSP8	880-3p	880-3p	138-5p	7i-5p				
DUSP15	124-3p							
Rps6ka3	411-5p	291a-3p						
Mef2c	31-5p	291a-3p						
Tnfrsf1a (TNFR1)	467e-5p	142a-3p						
TNF	30c-5p	19a-3p	19b-3p					
JunB	30c-5p	19a-3p	19b-3p	15b-5p				
Jun	19b-3p	19a-3p	15b-5p					
Fos	21a-5p							
RelA	9-5p	124-3p						
NFkb1	23b-3p	31-5p	15b-5p	23a-3p				
MAP3K7 (TGF-k)	10a-5p	23b-3p	7i-5p	124-3p				
Tgfbr1 (TGF-β-R1)	140-5p	880-3p	880-3p	124-3p				
Tgfbr2 (TGF-β-R2)	23b-3p							
TGF-beta	31-5p	141-3p						
ROCK1	31-5p	15b-3p	335-5p	29b-3p	141-3p			
ROCK2	142a-3p	23b-3p	19a-3p	19b-3p	9-5p	141-3p	23a-3p	124-3p
RhoA	138-5p	124-3p						
Col1a1	7i-5p	138-5p						
Col1a2	7i-5p							

Col4a1	7i-5p	138-5p						
Col4a2	7i-5p	138-5p						
Col5a1	31-5p							
Col5a2	7i-5p							
Fn1	144-3p							
Itgbb3 (integrina)	29a-3p	23b-3p	29b-3p	23a-5p				
Itgb8	214-3p	291a-3p						
VEGF	29a-3p	15b-5p	23b-3p	29b-3p	23a-5p	7i-5p		
HIF-1α	144-3p	29a-3p	21-5p					
Egrf	10b-5p	142a-3p	29b-3p	138-5p	23a-5p	7i-5p		
Ifngr (IFN-γ)	29a-3p	23b-3p						
SOCS1	19b-3p	7i-5p						
SOCS3	29a-3p	140-5p	19b-3p					
Sqstm1	144-3p	23b-3p	23a-5p					
IL1r1	335-5p	29b-3p						
IL-6	142-3p	7i-5p						
IL-6r	23b-3p	31-5p	23a-5p					
STAT1	23b-3p	19b-3p	15b-5p	19a-3p	15b-5p			
STAT2	9-5p	19b-3p	15b-5p	19a-3p	15b-5p			
STAT3	21-5p	31-5p	7i-5p					
IRF9	23b-3p							
PiK3ca	21a-5p							
PiK3r1 reg. sub. α	15b-3p	335-5p	29a-3p	31-5p	138-5p			
PiK3r3 reg. sub. γ	19a-3p	335-5p						
CAMK4	29a-3p	29b-3p	138-5p					
Ppp1cb	141-3p	29b-3p						
Ppp3ca	31-5p	7i-5p						
Ppp3cb	140-5p	15b-5p	29b-3p					
Ppp3r1-2	29a-3p	335-5p	31-5p	29b-3p	141-3p			
CALMG	411-5p							
CALM2	880-3p	880-3p						
GSK3β	199-5p	144-3p	880-3p	880-3p	124-3p			
DYRK1 -2	295-3p	880-3p	295-3p	880-3p				
OSCAR	124-3p							
MITF	19b-3p	19a-3p	144-3p	124-3p				
TRAF6	19b-3p	19a-3p	146-3p	15b-5p	880-3p	880-3p	124-3p	
NFATc2	214-3p	19a-3p	19b-3p	22-3p	124-3p			
NFATc1	124-3p							
ATP6Voa1	214-3p							
Ncf1 (NAPDH oxidase factor)	425-5p							
SMAD2	31-5p	880-3p	295-3p	880-3p	295-3p			
SMAD3	880-3p	880-3p	23a-3p					
SMAD4	214-3p	31-5p						
COLOR LEGEND	Group 1	Group 2	Group 3	Group 4	Inside the threshold			

S3A. Lists of genes involved in Regulation of actin cytoskeleton (04810) as predicted targets of the Group 1 miRNAs

	Gene Name	Gene Ensembl id		Gene Name	Gene Ensembl id
1.	Fgfr3	ENSMUSG00000054252	33.	Pik3cb	ENSMUSG00000032462
2.	Pxn	ENSMUSG00000029528	34.	Tiam1	ENSMUSG00000002489
3.	Apc	ENSMUSG00000005871	35.	Mylk	ENSMUSG00000022836
4.	Ppp1r12b	ENSMUSG00000073557	36.	Itgav	ENSMUSG00000027087
5.	Ppp1r12a	ENSMUSG00000019907	37.	Pikfyve	ENSMUSG00000025949
6.	Pik3r1	ENSMUSG00000041417	38.	Crk	ENSMUSG00000017776
7.	Pik3r3	ENSMUSG00000028698	39.	Diap2	ENSMUSG00000034480
8.	Myl12b	ENSMUSG00000034868	40.	Vcl	ENSMUSG00000021823
9.	Slc9a1	ENSMUSG00000028854	41.	Abi2	ENSMUSG00000026782
10.	Pdgfrb	ENSMUSG00000024620	42.	F2	ENSMUSG00000027249
11.	Wasl	ENSMUSG00000029684	43.	Ppp1cb	ENSMUSG00000014956
12.	Wasf2	ENSMUSG00000028868	44.	Myh9	ENSMUSG00000022443
13.	Fgf4	ENSMUSG00000050917	45.	Ssh2	ENSMUSG00000037926
14.	Gsn	ENSMUSG00000026879	46.	Arhgef6	ENSMUSG00000031133
15.	Arhgap35	ENSMUSG00000058230	47.	Araf	ENSMUSG00000001127
16.	Itgb3	ENSMUSG00000020689	48.	F2r	ENSMUSG00000048376
17.	Itgb7	ENSMUSG00000001281	49.	Arpc1b	ENSMUSG00000029622
18.	Pak1	ENSMUSG00000030774	50.	Itgb1	ENSMUSG00000025809
19.	Pfn1	ENSMUSG00000018293	51.	Itga7	ENSMUSG00000025348
20.	Cfl2	ENSMUSG00000062929	52.	Baiap2	ENSMUSG00000025372
21.	Crkl	ENSMUSG00000006134	53.	Gna13	ENSMUSG00000020611
22.	Egfr	ENSMUSG00000020122	54.	Enah	ENSMUSG00000022995
23.	Kras	ENSMUSG00000030265	55.	Pik3cd	ENSMUSG00000039936
24.	Braf	ENSMUSG00000002413	56.	Ssh1	ENSMUSG00000042121
25.	Rdx	ENSMUSG00000032050	57.	Itgb8	ENSMUSG00000025321
26.	Nras	ENSMUSG00000027852	58.	Myl9	ENSMUSG00000067818
27.	Rac1	ENSMUSG00000001847	59.	Pak2	ENSMUSG00000022781
28.	Rock2	ENSMUSG00000020580	60.	Fgf2	ENSMUSG00000037225
29.	Sos1	ENSMUSG00000024241	61.	Cdc42	ENSMUSG00000006699
30.	Actn4	ENSMUSG00000054808	62.	Pip4k2a	ENSMUSG00000026737
31.	Rras2	ENSMUSG00000055723	63.	Fgd1	ENSMUSG00000025265
32.	Actn1	ENSMUSG00000015143	64.	Arpc5	ENSMUSG00000008475

S3B. Lists of genes involved in Regulation of actin cytoskeleton (04810) as predicted targets of the Group 2 miRNAs

	Gene Name	Gene Ensembl id		Gene Name	Gene Ensembl id
1.	Vav2	ENSMUSG00000009621	48.	Braf	ENSMUSG00000002413
2.	Sos2	ENSMUSG00000034801	49.	Rdx	ENSMUSG00000032050
3.	Apc	ENSMUSG00000005871	50.	Nckap1	ENSMUSG00000027002
4.	Fgf10	ENSMUSG00000021732	51.	Nras	ENSMUSG00000027852
5.	Pip5k1c	ENSMUSG00000034902	52.	Rac1	ENSMUSG00000001847
6.	Pip4k2b	ENSMUSG00000018547	53.	Rock2	ENSMUSG00000020580
7.	Pik3r1	ENSMUSG00000041417	54.	Sos1	ENSMUSG00000024241
8.	Pik3r3	ENSMUSG00000028698	55.	Itga8	ENSMUSG00000026768
9.	Slc9a1	ENSMUSG00000028854	56.	Mras	ENSMUSG00000032470
10.	Pdgfrb	ENSMUSG00000024620	57.	Itgb2	ENSMUSG00000000290
11.	Arpc5l	ENSMUSG00000026755	58.	Rras2	ENSMUSG00000055723
12.	Msn	ENSMUSG00000031207	59.	Actn1	ENSMUSG00000015143
13.	Wasl	ENSMUSG00000029684	60.	Pik3cb	ENSMUSG00000032462
14.	Diap3	ENSMUSG00000022021	61.	Tiam1	ENSMUSG00000002489
15.	Wasf2	ENSMUSG00000028868	62.	Arpc1a	ENSMUSG00000029621
16.	Vav3	ENSMUSG00000033721	63.	Fgf18	ENSMUSG00000057967
17.	Map2k1	ENSMUSG00000004936	64.	Itgav	ENSMUSG00000027087
18.	Itga6	ENSMUSG00000027111	65.	Pikfyve	ENSMUSG00000025949
19.	Fgf7	ENSMUSG00000027208	66.	Crk	ENSMUSG00000017776
20.	Ezr	ENSMUSG00000052397	67.	Diap2	ENSMUSG00000034480
21.	Gsn	ENSMUSG00000026879	68.	Vcl	ENSMUSG00000021823
22.	Rock1	ENSMUSG00000024290	69.	Abi2	ENSMUSG00000026782
23.	Arhgap35	ENSMUSG00000058230	70.	Ppp1cb	ENSMUSG00000014956
24.	Fgf12	ENSMUSG00000022523	71.	Myh9	ENSMUSG00000022443
25.	Itgb3	ENSMUSG00000020689	72.	Arhgef7	ENSMUSG00000031511
26.	Itgb7	ENSMUSG00000001281	73.	Actg1	ENSMUSG00000062825
27.	Pak1	ENSMUSG00000030774	74.	Ssh2	ENSMUSG00000037926
28.	Myh10	ENSMUSG00000020900	75.	Arhgef6	ENSMUSG00000031133
29.	Bdkrb1	ENSMUSG00000041347	76.	Itga3	ENSMUSG00000001507
30.	Itga5	ENSMUSG00000000555	77.	Fgf9	ENSMUSG00000021974
31.	Pfn1	ENSMUSG00000018293	78.	F2r	ENSMUSG00000048376
32.	Src	ENSMUSG00000027646	79.	Arpc1b	ENSMUSG00000029622
33.	Cfl2	ENSMUSG00000062929	80.	Fgfr2	ENSMUSG00000030849
34.	Ppp1r12c	ENSMUSG00000019254	81.	Itgb1	ENSMUSG00000025809
35.	Actb	ENSMUSG00000029580	82.	Gna13	ENSMUSG00000020611
36.	Itga11	ENSMUSG00000032243	83.	Enah	ENSMUSG00000022995
37.	Pdgfd	ENSMUSG00000032006	84.	Itgb8	ENSMUSG00000025321
38.	Diap1	ENSMUSG00000024456	85.	Mylk4	ENSMUSG00000044951
39.	Itga1	ENSMUSG00000042284	86.	Pak2	ENSMUSG00000022781
40.	Arhgef12	ENSMUSG00000059495	87.	Cdc42	ENSMUSG00000006699
41.	Dock1	ENSMUSG00000058325	88.	Iqgap2	ENSMUSG00000021676
42.	Egfr	ENSMUSG00000020122	89.	Cyfip2	ENSMUSG00000020340
43.	Pfn2	ENSMUSG00000027805	90.	Pip4k2a	ENSMUSG00000026737
44.	Iqgap1	ENSMUSG00000030536	91.	Pak4	ENSMUSG00000030602
45.	Fgf13	ENSMUSG00000031137	92.	Limk2	ENSMUSG00000020451
46.	Kras	ENSMUSG00000030265	93.	Arpc5	ENSMUSG00000008475
47.	Pip5k1b	ENSMUSG00000024867			

S3C. Lists of genes involved in Regulation of actin cytoskeleton (04810) as predicted targets of the Group 4 miRNAs.

	Gene Name	Gene Ensembl id		Gene Name	Gene Ensembl id
1.	Apc	ENSMUSG00000005871	30.	Sos1	ENSMUSG00000024241
2.	Fgf10	ENSMUSG00000021732	31.	Itgb2	ENSMUSG00000000290
3.	Pip5k1c	ENSMUSG00000034902	32.	Rras2	ENSMUSG00000055723
4.	Pip4k2b	ENSMUSG00000018547	33.	Apc2	ENSMUSG00000020135
5.	Ppp1r12a	ENSMUSG00000019907	34.	Actn1	ENSMUSG00000015143
6.	Pik3r1	ENSMUSG00000041417	35.	Tiam1	ENSMUSG00000002489
7.	Bcar1	ENSMUSG00000031955	36.	Crk	ENSMUSG00000017776
8.	Slc9a1	ENSMUSG00000028854	37.	Diap2	ENSMUSG00000034480
9.	Pdgfrb	ENSMUSG00000024620	38.	Vcl	ENSMUSG00000021823
10.	Wasl	ENSMUSG00000029684	39.	Pip4k2c	ENSMUSG00000025417
11.	Cfl1	ENSMUSG00000056201	40.	Ppp1cb	ENSMUSG00000014956
12.	Wasf2	ENSMUSG00000028868	41.	Myh9	ENSMUSG00000022443
13.	Ptk2	ENSMUSG00000022607	42.	Ssh2	ENSMUSG00000037926
14.	Ezr	ENSMUSG00000052397	43.	Araf	ENSMUSG00000001127
15.	Itgb3	ENSMUSG00000020689	44.	Fgf9	ENSMUSG00000021974
16.	Pak1	ENSMUSG00000030774	45.	Rac2	ENSMUSG00000033220
17.	Arhgef4	ENSMUSG00000037509	46.	Gna13	ENSMUSG00000020611
18.	Rhoa	ENSMUSG00000007815	47.	Itgb8	ENSMUSG00000025321
19.	Pfn1	ENSMUSG00000018293	48.	Pak2	ENSMUSG00000022781
20.	Src	ENSMUSG00000027646	49.	Fgf2	ENSMUSG00000037225
21.	Cfl2	ENSMUSG00000062929	50.	Cdc42	ENSMUSG00000006699
22.	Crkl	ENSMUSG00000006134	51.	Cyfip2	ENSMUSG00000020340
23.	Arhgef12	ENSMUSG00000059495	52.	Pip4k2a	ENSMUSG00000026737
24.	Egfr	ENSMUSG00000020122	53.	Rras	ENSMUSG00000038387
25.	Kras	ENSMUSG00000030265	54.	Itgal	ENSMUSG00000030830
26.	Rdx	ENSMUSG00000032050	55.	Pak4	ENSMUSG00000030602
27.	Nckap1	ENSMUSG00000027002	56.	Limk2	ENSMUSG00000020451
28.	Nras	ENSMUSG00000027852	57.	Arpc5	ENSMUSG00000008475
29.	Rock2	ENSMUSG00000020580			

S4A. Lists of genes involved in ECM-receptor interaction (04512) as predicted targets of the Group 3 miRNAs.

	Gene Name	Gene Ensembl id
1.	Col3a1	ENSMUSG00000026043
2.	Itgb4	ENSMUSG00000020758
3.	Col6a3	ENSMUSG00000048126
4.	Itgb3	ENSMUSG00000020689
5.	Itgb7	ENSMUSG00000001281
6.	Thbs2	ENSMUSG00000023885
7.	Fn1	ENSMUSG00000026193
8.	Col5a2	ENSMUSG00000026042
9.	Sdc1	ENSMUSG00000020592
10.	Hmmr	ENSMUSG00000020330
11.	Col5a3	ENSMUSG00000004098
12.	Lamc1	ENSMUSG00000026478
13.	Col4a1	ENSMUSG00000031502
14.	Tnc	ENSMUSG00000028364
15.	Itgb1	ENSMUSG00000025809
16.	Col11a1	ENSMUSG00000027966
17.	Cd44	ENSMUSG00000005087
18.	Col6a2	ENSMUSG00000020241
19.	Lama5	ENSMUSG00000015647
20.	Itgb8	ENSMUSG00000025321
21.	Col4a2	ENSMUSG00000031503
22.	Col6a1	ENSMUSG00000001119
23.	Npnt	ENSMUSG00000040998

S5A. Lists of genes involved in Focal adhesion (04510) as predicted targets of the Group 1 miRNAs.

	Gene Name	Gene Ensembl id		Gene Name	Gene Ensembl id
1.	Pxn	ENSMUSG00000029528	28.	Arhgap5	ENSMUSG00000035133
2.	Ppp1r12b	ENSMUSG00000073557	29.	Rap1b	ENSMUSG00000052681
3.	Ppp1r12a	ENSMUSG00000019907	30.	Bcl2	ENSMUSG00000057329
4.	Flt1	ENSMUSG00000029648	31.	Actn4	ENSMUSG00000054808
5.	Pik3r1	ENSMUSG00000041417	32.	Actn1	ENSMUSG00000015143
6.	Pik3r3	ENSMUSG00000028698	33.	Pik3cb	ENSMUSG00000032462
7.	Gsk3b	ENSMUSG00000022812	34.	Mylk	ENSMUSG00000022836
8.	Myl12b	ENSMUSG00000034868	35.	Ccnd2	ENSMUSG00000000184
9.	Pdgfrb	ENSMUSG00000024620	36.	Itgav	ENSMUSG00000027087
10.	Rap1a	ENSMUSG00000068798	37.	Crk	ENSMUSG00000017776
11.	Tnr	ENSMUSG00000015829	38.	Prkca	ENSMUSG00000050965
12.	Birc3	ENSMUSG00000032000	39.	Vcl	ENSMUSG00000021823
13.	Arhgap35	ENSMUSG00000058230	40.	Ppp1cb	ENSMUSG00000014956
14.	Igf1	ENSMUSG00000020053	41.	Lamc1	ENSMUSG00000026478
15.	Itgb3	ENSMUSG00000020689	42.	Fyn	ENSMUSG00000019843
16.	Itgb7	ENSMUSG00000001281	43.	Itgb1	ENSMUSG00000025809
17.	Col1a1	ENSMUSG00000001506	44.	Itga7	ENSMUSG00000025348
18.	Pak1	ENSMUSG00000030774	45.	Pten	ENSMUSG00000013663
19.	Shc1	ENSMUSG00000042626	46.	Pik3cd	ENSMUSG00000039936
20.	Crkl	ENSMUSG00000006134	47.	Itgb8	ENSMUSG00000025321
21.	Egfr	ENSMUSG00000020122	48.	Myl9	ENSMUSG00000067818
22.	Braf	ENSMUSG00000002413	49.	Pak2	ENSMUSG00000022781
23.	Mapk9	ENSMUSG00000020366	50.	Col4a2	ENSMUSG00000031503
24.	Parva	ENSMUSG00000030770	51.	Cdc42	ENSMUSG00000006699
25.	Rac1	ENSMUSG00000001847	52.	Cav2	ENSMUSG00000000058
26.	Rock2	ENSMUSG00000020580	53.	Mapk8	ENSMUSG00000021936
27.	Sos1	ENSMUSG00000024241			

S5B. Lists of genes involved in Focal adhesion (04510) as predicted targets of the Group 2 miRNAs.

	Gene Name	Gene Ensembl id		Gene Name	Gene Ensembl id
1.	Vav2	ENSMUSG00000009621	46.	Parva	ENSMUSG00000030770
2.	Sos2	ENSMUSG00000034801	47.	Ccnd1	ENSMUSG00000070348
3.	Lama4	ENSMUSG00000019846	48.	Rac1	ENSMUSG00000001847
4.	Pip5k1c	ENSMUSG00000034902	49.	Rock2	ENSMUSG00000020580
5.	Flt1	ENSMUSG00000029648	50.	Sos1	ENSMUSG00000024241
6.	Pik3r1	ENSMUSG00000041417	51.	Arhgap5	ENSMUSG00000035133
7.	Ibsp	ENSMUSG00000029306	52.	Rap1b	ENSMUSG00000052681
8.	Pdpk1	ENSMUSG00000024122	53.	Itga8	ENSMUSG00000026768
9.	Jun	ENSMUSG00000052684	54.	Bcl2	ENSMUSG00000057329
10.	Pik3r3	ENSMUSG00000028698	55.	Actn1	ENSMUSG00000015143
11.	Gsk3b	ENSMUSG00000022812	56.	Pik3cb	ENSMUSG00000032462
12.	Pdgfrb	ENSMUSG00000024620	57.	Col24a1	ENSMUSG00000028197
13.	Kdr	ENSMUSG00000062960	58.	Ccnd2	ENSMUSG00000000184
14.	Rap1a	ENSMUSG00000068798	59.	Itgav	ENSMUSG00000027087
15.	Col3a1	ENSMUSG00000026043	60.	Crk	ENSMUSG00000017776
16.	Spp1	ENSMUSG00000029304	61.	Prkca	ENSMUSG00000050965
17.	Vav3	ENSMUSG00000033721	62.	Col1a2	ENSMUSG00000029661
18.	Map2k1	ENSMUSG00000004936	63.	Vcl	ENSMUSG00000021823
19.	Itga6	ENSMUSG00000027111	64.	Ppp1cb	ENSMUSG00000014956
20.	Thbs1	ENSMUSG00000040152	65.	Actg1	ENSMUSG00000062825
21.	Rock1	ENSMUSG00000024290	66.	Elk1	ENSMUSG00000009406
22.	Birc3	ENSMUSG00000032000	67.	Lamc1	ENSMUSG00000026478
23.	Arhgap35	ENSMUSG00000058230	68.	Itga3	ENSMUSG00000001507
24.	Igf1	ENSMUSG00000020053	69.	Fyn	ENSMUSG00000019843
25.	Itgb3	ENSMUSG00000020689	70.	Tnc	ENSMUSG00000028364
26.	Itgb7	ENSMUSG00000001281	71.	Itgb1	ENSMUSG00000025809
27.	Grb2	ENSMUSG00000059923	72.	Col11a1	ENSMUSG00000027966
28.	Pak1	ENSMUSG00000030774	73.	Vtn	ENSMUSG00000017344
29.	Shc1	ENSMUSG00000042626	74.	Pten	ENSMUSG00000013663
30.	Col27a1	ENSMUSG00000045672	75.	Flnc	ENSMUSG00000068699
31.	Itga5	ENSMUSG00000000555	76.	Col6a2	ENSMUSG00000020241
32.	Src	ENSMUSG00000027646	77.	Erbb2	ENSMUSG00000062312
33.	Rasgrf1	ENSMUSG00000032356	78.	Itgb8	ENSMUSG00000025321
34.	Ppp1r12c	ENSMUSG00000019254	79.	Mylk4	ENSMUSG00000044951
35.	Actb	ENSMUSG00000029580	80.	Pak2	ENSMUSG00000022781
36.	Itga11	ENSMUSG00000032243	81.	Met	ENSMUSG00000009376
37.	Pdgfd	ENSMUSG00000032006	82.	Col4a2	ENSMUSG00000031503
38.	Diap1	ENSMUSG00000024456	83.	Cdc42	ENSMUSG00000006699
39.	Vegfa	ENSMUSG00000023951	84.	Col5a1	ENSMUSG00000026837
40.	Itga1	ENSMUSG00000042284	85.	Shc4	ENSMUSG00000035109
41.	Dock1	ENSMUSG00000058325	86.	Tln1	ENSMUSG00000028465
42.	Egfr	ENSMUSG00000020122	87.	Cav2	ENSMUSG00000000058
43.	Thbs2	ENSMUSG00000023885	88.	Mapk8	ENSMUSG00000021936
44.	Braf	ENSMUSG00000002413	89.	Pak4	ENSMUSG00000030602
45.	Mapk9	ENSMUSG00000020366			

S5C. Lists of genes involved in Focal adhesion (04510) as predicted targets of the Group 3 miRNAs.

	Gene Name	Gene Ensembl id		Gene Name	Gene Ensembl id
1.	Sos2	ENSMUSG00000034801	28.	Col5a2	ENSMUSG00000026042
2.	Ppp1r12b	ENSMUSG00000073557	29.	Pik3cb	ENSMUSG00000032462
3.	Pik3r1	ENSMUSG00000041417	30.	Col5a3	ENSMUSG00000004098
4.	Parvb	ENSMUSG00000022438	31.	Pdgfb	ENSMUSG00000000489
5.	Pdpk1	ENSMUSG00000024122	32.	Ccnd2	ENSMUSG00000000184
6.	Gsk3b	ENSMUSG00000022812	33.	Crk	ENSMUSG00000017776
7.	Pdgfrb	ENSMUSG00000024620	34.	Prkca	ENSMUSG00000050965
8.	Rap1a	ENSMUSG00000068798	35.	Ppp1cb	ENSMUSG00000014956
9.	Col3a1	ENSMUSG00000026043	36.	Elk1	ENSMUSG00000009406
10.	Flnb	ENSMUSG00000025278	37.	Lamc1	ENSMUSG00000026478
11.	Itgb4	ENSMUSG00000020758	38.	Col4a1	ENSMUSG00000031502
12.	Col6a3	ENSMUSG00000048126	39.	Fyn	ENSMUSG00000019843
13.	Rock1	ENSMUSG00000024290	40.	Tnc	ENSMUSG00000028364
14.	Birc3	ENSMUSG00000032000	41.	Itgb1	ENSMUSG00000025809
15.	Igf1	ENSMUSG00000020053	42.	Col11a1	ENSMUSG00000027966
16.	Itgb3	ENSMUSG00000020689	43.	Pten	ENSMUSG00000013663
17.	Itgb7	ENSMUSG00000001281	44.	Col6a2	ENSMUSG00000020241
18.	Hgf	ENSMUSG00000028864	45.	Erbb2	ENSMUSG00000062312
19.	Vegfa	ENSMUSG00000023951	46.	Lama5	ENSMUSG00000015647
20.	Egfr	ENSMUSG00000020122	47.	Itgb8	ENSMUSG00000025321
21.	Thbs2	ENSMUSG00000023885	48.	Col4a2	ENSMUSG00000031503
22.	Braf	ENSMUSG00000002413	49.	Cdc42	ENSMUSG00000006699
23.	Fn1	ENSMUSG00000026193	50.	Shc4	ENSMUSG00000035109
24.	Rock2	ENSMUSG00000020580	51.	Col6a1	ENSMUSG00000001119
25.	Sos1	ENSMUSG00000024241	52.	Cav2	ENSMUSG00000000058
26.	Arhgap5	ENSMUSG00000035133	53.	Mapk8	ENSMUSG00000021936
27.	Rap1b	ENSMUSG00000052681			

S5D. Lists of genes involved in Focal adhesion (04510) as predicted targets of the Group 4 miRNAs.

	Gene Name	Gene Ensembl id		Gene Name	Gene Ensembl id
1.	Pip5k1c	ENSMUSG00000034902	27.	Igf1r	ENSMUSG00000005533
2.	Ppp1r12a	ENSMUSG00000019907	28.	Rock2	ENSMUSG00000020580
3.	Flt1	ENSMUSG00000029648	29.	Sos1	ENSMUSG00000024241
4.	Pik3r1	ENSMUSG00000041417	30.	Arhgap5	ENSMUSG00000035133
5.	Pdpk1	ENSMUSG00000024122	31.	Bcl2	ENSMUSG00000057329
6.	Bcar1	ENSMUSG00000031955	32.	Actn1	ENSMUSG00000015143
7.	Pdgfrb	ENSMUSG00000024620	33.	Ccnd2	ENSMUSG00000000184
8.	Kdr	ENSMUSG00000062960	34.	Crk	ENSMUSG00000017776
9.	Spp1	ENSMUSG00000029304	35.	Prkca	ENSMUSG00000050965
10.	Tnr	ENSMUSG00000015829	36.	Col1a2	ENSMUSG00000029661
11.	Ptk2	ENSMUSG00000022607	37.	Vcl	ENSMUSG00000021823
12.	Thbs1	ENSMUSG00000040152	38.	Ppp1cb	ENSMUSG00000014956
13.	Reln	ENSMUSG00000042453	39.	Lama1	ENSMUSG00000032796
14.	Birc3	ENSMUSG00000032000	40.	Col4a1	ENSMUSG00000031502
15.	Igf1	ENSMUSG00000020053	41.	Fyn	ENSMUSG00000019843
16.	Itgb3	ENSMUSG00000020689	42.	Rac2	ENSMUSG00000033220
17.	Col1a1	ENSMUSG00000001506	43.	Pten	ENSMUSG00000013663
18.	Pak1	ENSMUSG00000030774	44.	Itgb8	ENSMUSG00000025321
19.	Rhoa	ENSMUSG00000007815	45.	Pak2	ENSMUSG00000022781
20.	Src	ENSMUSG00000027646	46.	Met	ENSMUSG00000009376
21.	Rasgrf1	ENSMUSG00000032356	47.	Lamb1	ENSMUSG00000002900
22.	Vegfa	ENSMUSG00000023951	48.	Col4a2	ENSMUSG00000031503
23.	Crkl	ENSMUSG00000006134	49.	Cdc42	ENSMUSG00000006699
24.	Egfr	ENSMUSG00000020122	50.	Shc4	ENSMUSG00000035109
25.	Mapk9	ENSMUSG00000020366	51.	Tln1	ENSMUSG00000028465
26.	Ccnd1	ENSMUSG00000070348	52.	Pak4	ENSMUSG00000030602

S6A. Lists of genes involved in Osteoclast differentiation (04380) as predicted targets of the Group 1 miRNA.

	Gene Name	Gene Ensembl id
1.	Mapk14	ENSMUSG00000053436
2.	Pik3r1	ENSMUSG00000041417
3.	Pik3r3	ENSMUSG00000028698
4.	Cybb	ENSMUSG00000015340
5.	Stat2	ENSMUSG00000040033
6.	Tnfrsf1a	ENSMUSG00000030341
7.	Socs3	ENSMUSG00000053113
8.	Ifngr1	ENSMUSG00000020009
9.	Fhl2	ENSMUSG00000008136
10.	Map2k6	ENSMUSG00000020623
11.	Itgb3	ENSMUSG00000020689
12.	Creb1	ENSMUSG00000025958
13.	Il1r1	ENSMUSG00000026072
14.	Junb	ENSMUSG00000052837
15.	Sqstm1	ENSMUSG00000015837
16.	Ifngr2	ENSMUSG00000022965
17.	Lcp2	ENSMUSG00000002699
18.	Ppp3cb	ENSMUSG00000021816
19.	Mapk9	ENSMUSG00000020366
20.	Tgfbr2	ENSMUSG00000032440
21.	Rac1	ENSMUSG00000001847
22.	Camk4	ENSMUSG00000038128
23.	Jak1	ENSMUSG00000028530
24.	Mitf	ENSMUSG00000035158
25.	Pik3cb	ENSMUSG00000032462
26.	Tnf	ENSMUSG00000024401
27.	Socs1	ENSMUSG00000038037
28.	Fosl2	ENSMUSG00000029135
29.	Fyn	ENSMUSG00000019843
30.	Ppp3r1	ENSMUSG00000033953
31.	Ppp3ca	ENSMUSG00000028161
32.	Calcr	ENSMUSG00000023964
33.	Tnfsf11	ENSMUSG00000022015
34.	Pik3cd	ENSMUSG00000039936
35.	Tab2	ENSMUSG00000015755
36.	Mapk8	ENSMUSG00000021936

S6B. Lists of genes involved in Osteoclast differentiation (04380) as predicted targets of the Group 2 miRNA.

	Gene Name	Gene Ensembl id		Gene Name	Gene Ensembl id
1.	Tgfb2	ENSMUSG00000039239	26.	Tgfbr2	ENSMUSG00000032440
2.	Mapk14	ENSMUSG00000053436	27.	Rac1	ENSMUSG00000001847
3.	Irf9	ENSMUSG00000002325	28.	Camk4	ENSMUSG00000038128
4.	Pik3r1	ENSMUSG00000041417	29.	Jak1	ENSMUSG00000028530
5.	Jun	ENSMUSG00000052684	30.	Map2k7	ENSMUSG00000002948
6.	Pik3r3	ENSMUSG00000028698	31.	Ifng	ENSMUSG00000055170
7.	Stat2	ENSMUSG00000040033	32.	Mitf	ENSMUSG00000035158
8.	Fosl1	ENSMUSG00000024912	33.	Pik3cb	ENSMUSG00000032462
9.	Stat1	ENSMUSG00000026104	34.	Tnf	ENSMUSG00000024401
10.	Socs3	ENSMUSG00000053113	35.	Nfkb1	ENSMUSG00000028163
11.	Ifngr1	ENSMUSG00000020009	36.	Socs1	ENSMUSG00000038037
12.	Map2k1	ENSMUSG00000004936	37.	Fosl2	ENSMUSG00000029135
13.	Fos	ENSMUSG00000021250	38.	Fyn	ENSMUSG00000019843
14.	Map2k6	ENSMUSG00000020623	39.	Ppp3r1	ENSMUSG00000033953
15.	Ncf1	ENSMUSG00000015950	40.	Traf6	ENSMUSG00000027164
16.	Itgb3	ENSMUSG00000020689	41.	Csf1	ENSMUSG00000014599
17.	Creb1	ENSMUSG00000025958	42.	Calcr	ENSMUSG00000023964
18.	Junb	ENSMUSG00000052837	43.	Tnfsf11	ENSMUSG00000022015
19.	Grb2	ENSMUSG00000059923	44.	Cyld	ENSMUSG00000036712
20.	Sqstm1	ENSMUSG00000015837	45.	Mapk11	ENSMUSG00000053137
21.	Ifngr2	ENSMUSG00000022965	46.	Sirpa	ENSMUSG00000037902
22.	Lcp2	ENSMUSG00000002699	47.	Map3k7	ENSMUSG00000028284
23.	Tgfbr1	ENSMUSG00000007613	48.	Nfatc2	ENSMUSG00000027544
24.	Ifnar2	ENSMUSG00000022971	49.	Tab2	ENSMUSG00000015755
25.	Mapk9	ENSMUSG00000020366	50.	Mapk8	ENSMUSG00000021936

Figure S2. Western blot with two independent biological samples (1° and 2° experiments), detected with NFATc1, ERK1/2, p-ERK1/2 and β-actin antibodies. Proteins were visualized using an Odyssey Infrared Imaging System (LI-COR Lincoln) according to the manufacturer's instructions. Fluorescence quantification of proteins was performed with the application software, version 3.0 (LI-COR, Biosciences) and values were shown as Integrated Intensity.

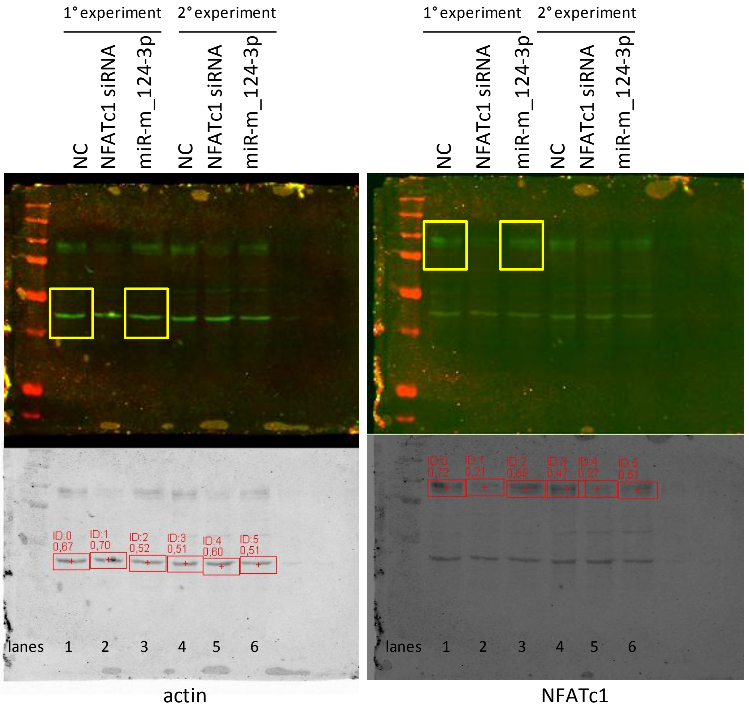

NFATc1				
lanes	1	3	4	6
	NC	mimic	NC	mimic
NFATc1	0.72	0.65	0.47	0.51
actin	0.67	0.52	0.51	0.51
NFATc1/actin	1.07	1.25	0.92	1.00
**mimic/NC**	**1**	**1.16**	**1**	**1.09**

mean of the values corresponding to the lanes:

		1 and 4	3 and 6
**mimic/NC**	**mean**	**1.00**	**1.12**
	**dev st**	**0.00**	**0.06**

NC, negative control
mimic, miR-m_124-3p

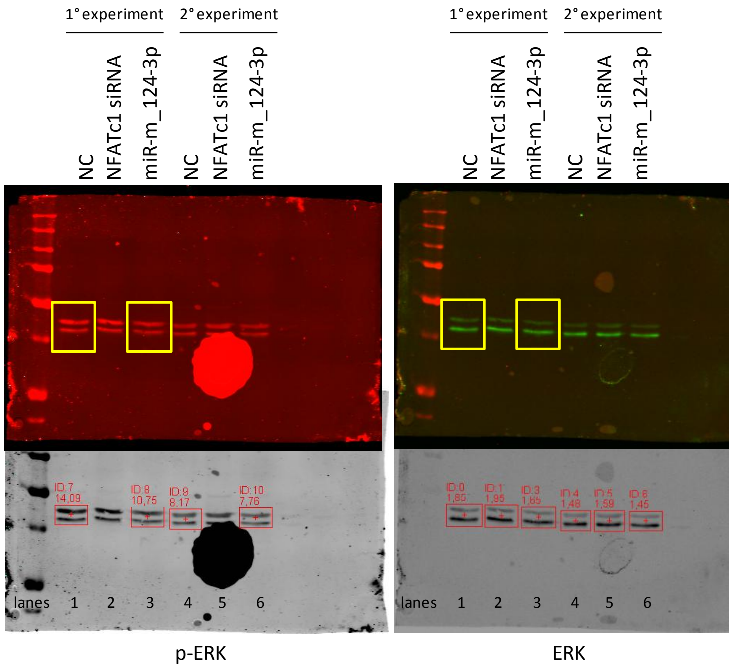

p-ERK				
lanes	1	3	4	6
	NC	mimic	NC	mimic
p-ERK	14.09	10.75	8.17	7.76
ERK	1.85	1.65	1.48	1.45
P-ERK/ERK	7.62	6.52	5.52	5.35
**mimic/NC**	**1**	**0.86**	**1**	**0.97**
actin	0.67	0.52	0.51	0.51
ERK/actin	2.76	3.17	2.90	2.84
p-ERK/actin	21.03	20.67	16.02	15.22
P-ERK act/ERK act	7.62	6.52	5.52	5.35
**mimic/NC**	**1**	**0.86**	**1**	**0.97**

mean of the values corresponding to the lanes:

		1 and 4	3 and 6
**mimic/NC**	**mean**	**1**	**0.91**
	**dev st**	**0**	**0.08**

NC, negative control
mimic, miR-m_124-3p

Within the yellow frames, the bands used for Figure 5D
